# Post-Traumatic Stress Symptoms and Post-Traumatic Growth: Evidence from a Longitudinal Study following an Earthquake Disaster

**DOI:** 10.1371/journal.pone.0127241

**Published:** 2015-06-05

**Authors:** Jieling Chen, Xiao Zhou, Min Zeng, Xinchun Wu

**Affiliations:** School of Psychology, Beijing Normal University, Beijing, China; Central Institute of Mental Health, GERMANY

## Abstract

**Objective:**

The current longitudinal study aims to examine the bidirectional relationship between post-traumatic stress symptoms (PTSS) and post-traumatic growth (PTG).

**Method:**

One hundred twenty-two adults in the most severely affected area were investigated by self-report questionnaires at 12 months and 18 months after the Wenchuan Earthquake occurred in China.

**Results:**

The autoregressive cross-lagged structure equation analysis revealed that PTG at 12 months post-earthquake could negatively predict PTSS at 18 months post-earthquake above and beyond PTSS stability, whereas PTSS at 12 months post-earthquake could not significantly predict subsequent PTG. Moreover, PTG at 12 months post-earthquake could predict fewer subsequent intrusions, numbing and hyper-arousal symptoms but not avoidance symptoms.

**Conclusion:**

Growth can play a role in reducing long-term post-traumatic stress symptoms, and the implication of a positive perspective in post-trauma circumstance is discussed.

## Introduction

Post-traumatic stress disorder (PTSD) is a psychiatric disorder that can occur in people who have experienced (directly or indirectly) or witnessed a traumatic event. It includes symptoms such as intrusion, avoidance, numbing, and hyper-arousal [1]. Post-traumatic stress symptoms (PTSS) are often considered the most common negative psychological reactions in the aftermath of trauma [2,3].

Over the past decades, researchers have moved away from an exclusive focus on the negative aftermath following traumatic events. A growing body of studies document positive psychological changes after traumatic events [4,5]. Tedeschi and Calhoun [6] referred to this phenomenon as post-traumatic growth (PTG). It emphasizes the transformative quality of responding to traumatic events. The positive changes entail several domains, including perceived changes in self, a changed sense of relationship with others, and a changed philosophy of life.

PTSD and PTG are regarded as two distinct constructs [7–9]. Domains of growth are concerned with fundamental positive changes in schema and assumptive worlds, which is conceptually distinct from cognitive-emotional adjustment [7]. Therefore, PTG and PTSS can coexist in individuals. As such, an imperative issue is the relationship between them. Some researchers have addressed this issue by reviewing relevant studies [9,10]. However, the findings were not consistent: some studies found that the association between PTSS and PTG was not significant [11], and some studies reported a significant association, either in a positive manner [12] or in a negative manner [13]. Among the existing studies, a majority of them had cross-sectional designs. This is a crucial limitation, as the relationship between PTSS and PTG, either the predictive effect of PTSS on PTG, or the potential adaptive value of PTG, is suggested to emerge over a period of time [8,9]. Therefore, the cross-sectional designs fail to detect the relationships between the catalyst variable and the outcome variable, making it difficult to elucidate the nature of the association.

Although the longitudinal studies of the relationship between PTSS and PTG are relatively limited [14], from the preliminary evidence, possible paths might be indicated. A possible path is that initial PTSS elicit subsequent PTG. Tedeschi and Calhoun [8] have suggested that stress induced by traumatic events can stimulate the cognitive processing, and when it transforms into constructive processing, individuals can reconstruct the schema and assumptions, resulting in positive changes. For instance, a study among war veterans found that initial levels of PTSS had a positive effect on PTG five years later [15]. Another study had followed a sample of Israeli ex-prisoners of war over 17 years, and it revealed that PTSS measured at 18 years and 30 years since the war could positively predict PTG measured at 30 years and 35 years since the war, respectively [14]. Viewing PTG as the outcome of a psychological struggle post-trauma, PTSS are expected to play a positive role in the attainment of PTG.

On the other hand, an alternative path is that growth affects the subsequent distress. Davis, Nolen-Hoeksema and Larson [16] regard PTG as one construal of meaning, which would signify a benefit attribution to the question “what for?” In Taylor and Armor’s formulation [17], PTG has been considered as one form of self-enhancing appraisal, which can help to cope with a threat. Consistent with these findings, a study among 171 women suffering from sexual abuse revealed that those with higher levels of PTG two weeks after the events suffered from fewer PTSS 12 months after the events [13]. Another study among survivors of disasters (tornados, mass killing and plane crashes) indicated that perceived benefit 4–6 weeks post-disaster predicted fewer PTSS three years later [18]. In viewing PTG as a coping strategy, it is expected that initial PTG would predict fewer subsequent PTSS. However, it is suggested that the notion of the adaptive significance of PTG still needs to be tested further [9]. For one thing, a few studies found that PTSS and PTG were unrelated in the time course [19,20]. Additionally, when the PTG was found to be adaptive, unstandardized and unvalidated measures of growth were often used.

In summary, most of the existing longitudinal studies investigating the association of PTSS and growth have only examined the unidirectional effect of PTSS on PTG or PTG on PTSS. However, the unidirectional models fail to test the two possible paths concurrently, making it impossible to indicate the bidirectional relationship between PTSS and PTG. There is one exception in a sample of Israeli ex-prisoners of war who were followed for over 17 years and were found to have growth as a response to distress and not vice versa [14]. Nevertheless, whether this pattern can be applied to different circumstances still needs to be examined. For instance, in Dekel et al.’s study, all three assessment points were over 18 years since the war. As a majority of changes in the psychological adjustment occur during the first 2-year period following traumatic events [21,22], the bidirectional relationship between PTSS and PTG within this period should be examined further.

In the current study, we measure the PTSS and PTG among a group of adults who experienced a catastrophic earthquake that took place in China, the Wenchuan Earthquake, at two time points: 12 months and 18 months post-earthquake. The bidirectional relationship between PTSS and PTG during this time course will be explored by constructing autoregressive cross-lagged models.

## Methods

### Participants

The current study was carried out in the area most affected by Wenchuan Earthquake, including Wenchuan county and Mao county, among the survivors of the earthquake. The first survey was conducted from late May to early June in 2009 (T1), and 223 adults were surveyed by random cluster sampling. The follow-up survey was conducted from late November to early December in 2009 (T2), and 122 participants who had participated in the first survey were interviewed in the follow-up survey, with an attrition rate of 45%. The current study used data from 122 individuals who participated in the 12- and 18-month post-earthquake surveys. The demographic characteristics were shown in Table 1. The mean age of the study sample was 33.9 with a range from 23 to 43. Over half (63.1%) of the participants were trapped and 6.6% were injured in the earthquake. The houses of 59.8% participants were severely or totally destroyed in the earthquake. Nearly half (48.4%) of the participants were worried about being injured, and 58.2% were worried about dying during the earthquake. Two participants lost a parent whereas others did not suffer from death of a spouse, child, or parent. The excluded samples did not differ significantly from the included samples on the four subscales of PTSS at T1, the three subscales of PTG at T1, the percentage of injured, and the percentage of severe or total home destruction, the percentage of worrying about dying or being injured. The percentage of trapped did differ between the excluded and included samples, that is, the included sample (63.1%) reported a higher percentage of being trapped than the excluded sample (44.6%).

**Table 1:** Demographic Characteristics.

Variable	*n*	%
**Gender**		
Female	69	56.6
Male	53	43.4
**Exposure**		
Be trapped in earthquake	77	63.1
Be injured in earthquake	8	6.6
Death of parent	2	1.6
Death of spouse	0	0
Death of child	0	0
Home destruction (severe or total)	73	59.8
Worried about being injured	59	48.4
Worried about dying	71	58.2

### Measures

#### Post-traumatic stress symptoms

PTSS was measure by the Chinese Version of Impact of Event Scale-Revised (IES-R) in the current study. The scale was first developed by Weiss and Marmar [23] and was modified for application in a Chinese sample [24]. It consists of 22 items. Participants were asked to indicate how much they were distressed or bothered during the past seven days on a 5-point scale ranging from 0 (“not at all”) to 4 (“extremely”). The Chinese version of the IES-R demonstrated high reliability and concurrent validity compared with clinical diagnoses [24]. In the original scale, a three-factor structure was suggested in accordance with the DSM-IV [25], in which eight items measure intrusion symptoms, eight items measure avoidance/numbing symptoms, and six items measure hyper-arousal symptoms. However, the three-factor structure of PTSD as defined in the DSM-IV has been challenged in the past decade. For instance, King, D., Leskin, King, L., and Weather [26] proposed a four-factor model that splits the avoidance/numbing cluster into discrete clusters, and their construct distinctiveness was supported in empirical research [27–29]. Accordingly, in the DSM-V [1], the avoidance/numbing symptom cluster of the DSM-IV is split into two separate clusters. The new cluster, similar to the numbing cluster, is relabeled as “negative alterations in mood and cognition”. In the current study, a four-factor model as proposed by King et al. [26] was tested, with eight items loading on the intrusion factor, six items on the avoidance factor, two items on the numbing factor and six items on the hyper-arousal factor. Confirmatory factor analysis (CFA) was conducted using the data of 223 participants assessed at T1. The main results were as follows: *χ* ^*2*^/*df* = 2.25, *p*<0.01; RMSEA = 0.08; 90% Confidence Interval of RMSEA = [0.07, 0.08]; CFI = 0.89; TLI = 0.87. Thus, the construct of four symptom clusters was supported in the current study samples. The Cronbach’s alpha for the total scale was 0.93 at T1 and 0.94 at T2. The Cronbach’s alpha for the intrusion, avoidance, numbing and hyper-arousal symptoms were 0.88, 0.83, 0.50 and 0.85 at T1; 0.91, 0.86, 0.53 and 0.87 at T2, respectively.

#### Post-traumatic growth

PTG was measured by a modified version of the Post-traumatic Growth Inventory (PTGI) [30]. The original PTGI was developed by Tedeschi and Calhoun [31] for the assessment of positive changes following traumatic events. The inventory consists of 21 items, and each item is rated on a 6-point scale ranging from 0 (no change) to 5 (great change). The inventory was translated and revised by a research project among the survivors of Wenchuan earthquake [30]. Compared with the original scale, one item was added. The CFA results supported a model with three factors: perceived changes in self, a changed sense of relationship with others, and a changed philosophy of life. The fit indices were as follows: *χ* ^*2*^/*df* = 2.35, RMSEA = 0.07, CFI = 0.93. The Cronbach’s alpha for the total scale was 0.95 at T1 and 0.96 at T2. Cronbach’s alpha for perceived changes in self, a changed sense of relationship with others and a changed philosophy of life were 0.89, 0.89 and 0.72 at T1; 0.91, 0.91 and 0.78 at T2, respectively.

### Procedures and data analysis

From late May to early June in 2009, surveys were carried out in twelve primary and secondary schools among children and adolescents in two counties, including Wenchuan county and Mao county, which were most affected by the earthquake. Meanwhile, adults in those schools, mainly teachers, were surveyed by random sampling. The current study used data of adult participants. From late November to early December in 2009, follow-up surveys were carried out. Questionnaires were sent to each participant. We received the finished questionnaires in one to two weeks. This study was approved by the Ethics Review Committee of School of Psychology, Beijing Normal University and the principals of the participating schools. Written informed consent was obtained from each participant. It was emphasized in the written consent forms that we would protect the confidentiality of each participant, and each participant had the right to withdraw from the study at any time. Data collection was carried out by trained individuals with bachelor’s degrees in psychology.

Descriptive analyses were conducted to measure the levels of PTSS and PTG. Pearson correlations and partial correlations were calculated to examine the associations between PTSS and PTG, both cross-sectionally and longitudinally.

To examine the bidirectional relationships between PTSS and PTG, an autoregressive cross-lagged modeling strategy (ARCL) [32] was constructed. It allows for the simultaneous assessment of the stability of PTSS and PTG as well as cross-lagged paths both from initial PTSS to subsequent PTG and initial PTG to subsequent PTSS. Missing data were handled with full information maximum likelihood estimation. To evaluate the model fit, we used chi-square values, the comparative fit index (CFI), Tucker-Levis index (TLI), and the root mean square error of approximation (RMSEA). A CFI >0.9 suggested a model of good fit [33] and a RMSEA <0.08 suggested a model of adequate fit [34]. Statistical analyses were conducted using Mplus 7.0 software [35].

## Results

### Descriptive Statistics

The means, standard deviation, and inter-correlations between the main study variables are shown in Table 2. Huang et al. [24] suggested 35 was an appropriate cutoff indicative of PTSD in Chinese samples. According to this criterion, the current samples showed overall moderate PTSS symptoms at 12 and 18 months post-earthquake. Meanwhile, the mean score on overall PTG was moderately high at both time points. T1 PTSS and T2 PTSS, as well as T1 PTG and T2 PTG, yielded significant correlations, respectively, as higher levels of PTSS/PTG at 12 months post-earthquake were associated with the higher levels of PTSS/PTG at 18 months post-earthquake. Cross-sectional correlations between PTSS and PTG were not significant. Correlations between PTSS and PTG over two time-points were not significant. Due to the significant auto-correlation of PTSS and PTG, we explored the partial correlation between PTSS and PTG over two time-points: when controlling for the T1 PTG, the partial correlation between T1 PTSS and T2 PTG was not significant (*r* = 0.07, *p* > 0.05); but when controlling for the T1 PTSS, the partial correlation between T1 PTG and T2 PTSS was significantly negative (*r* = -0.25, *p* < 0.05).

**Table 2:** PTSS and PTG Descriptive Statistics.

	M±SD	1	2	3	4
**1. T1 PTSS**	31.69±17.27	-	-	(0.59**)	(0.07)
**2. T1 PTG**	61.54±22.33	0.16	-	-0.25*	0.31**
**3. T2 PTSS**	30.72±16.67	0.56**	-0.12	-	-0.03(0.06)
**4. T2 PTG**	62.86±20.79	0.12	0.33**	0.04	-

*Note:* *p < 0.05

**p < 0.01. Pearson correlations between variables are shown below the diagonal. Above the diagonal, the numbers in parenthesis refer to partial correlations between variables when controlling for T1 PTG, and the numbers without parenthesis refer to partial correlations between variables when controlling for T1 PTSS.

### Bidirectional relations between PTSS and PTG in a time course analysis

To examine the bidirectional relationships between PTSS and PTG over two time points, an autoregressive cross-lagged model (M1) was constructed. Specifically, the latent variable of PTSS was derived from the intrusion, avoidance, numbing and hyper-arousal, and latent variable of PTG was derived from perceived changes in self, a changed sense of relationship with others, and a changed philosophy of life. The model fit indices were as follows: χ^2^ (71) = 136.773, *p*<0.01; CFI = 0.951; TLI = 0.938; RMSEA = 0.087; 90% RMSEA CI [0.065, 0.109]. The RMSEA did not meet the suggested cut-off criteria, indicating the model did not fit particularly well.

**Fig 1:**
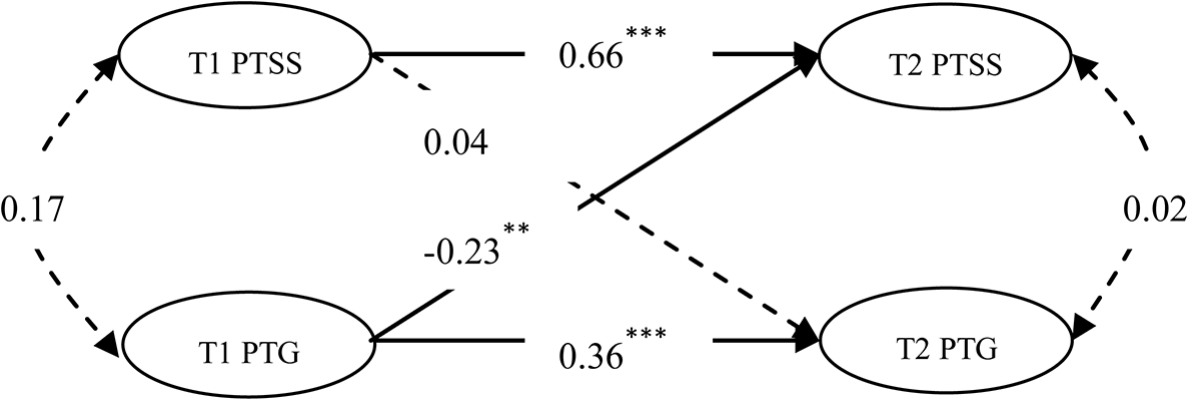
The Autoregressive Cross-lagged Model of PTSS and PTG (M2).

As examination of the modification indices signified the need for freeing error covariance between avoidance at T1 and avoidance at T2. When testing the model with covariance parameters (M2), the results indicated that the model fit the data well: χ^2^ (70) = 116.493, *p*<0.01; CFI = 0.966; TLI = 0.955; RMSEA = 0.074; 90% RMSEA CI [0.049, 0.097]. The M2 results are presented in Fig 1. Both PTSS and PTG demonstrated stability over time. The individuals with higher levels of PTSS/PTG at T1 tended to have higher levels of PTSS/PTG at T2. Moreover, the initial level of PTG at T1 could negatively predict the subsequent level of PTSS at T2 (*β* = -0.23), and the initial level of PTSS at T1 could not predict the subsequent level of PTG at T2 (*β* = 0.04).

**Fig 2:**
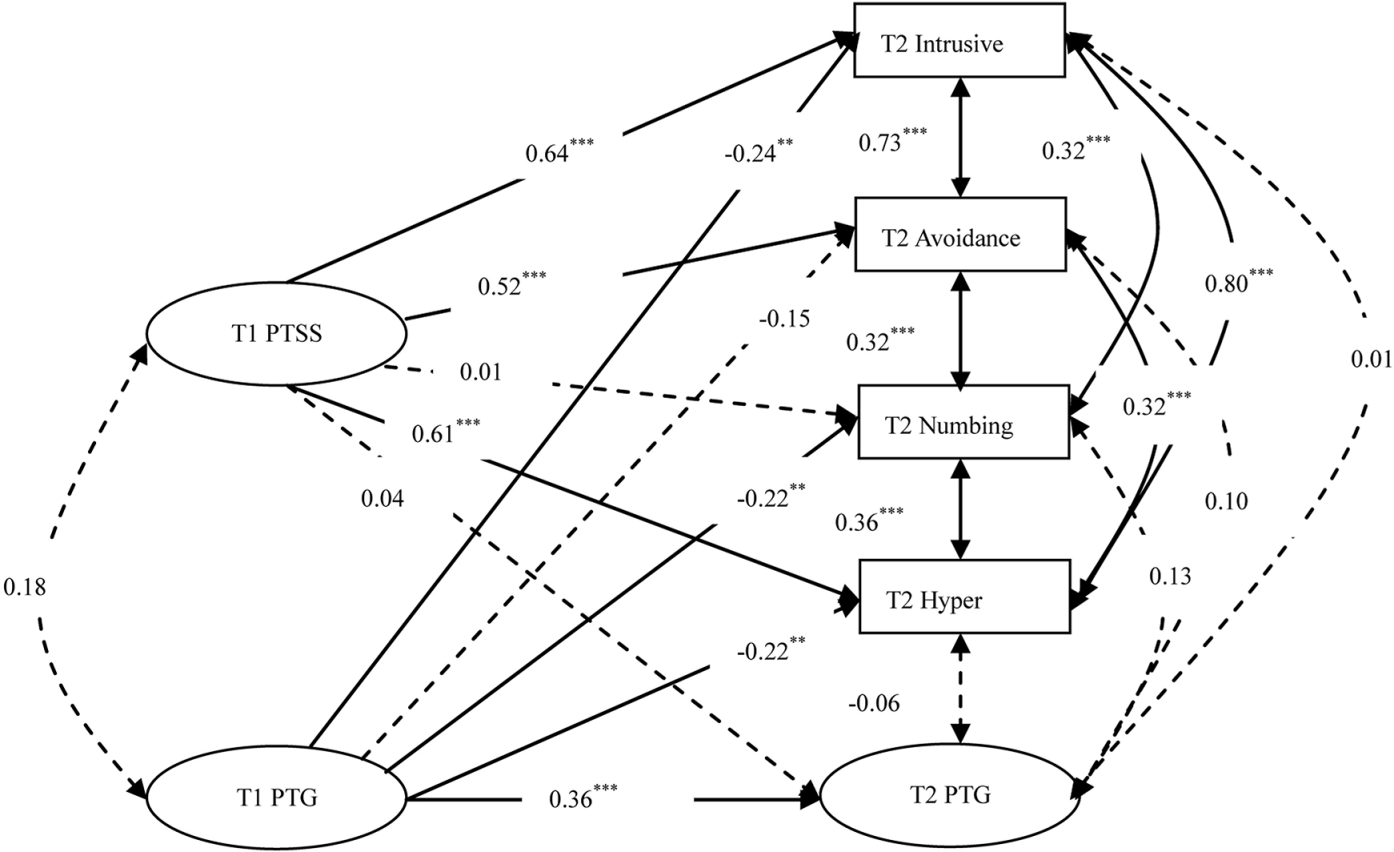
The Autoregressive Cross-lagged Model of PTG and PTSD Symptom Clusters (M3).

Next, we explore whether initial PTG could predict all subsequent PTSS clusters (intrusion, avoidance, numbing, and hyper-arousal) or only specific clusters of PTSS. We ran another ARCL model in which PTSS clusters at T2 were severed as outcomes of PTG at T1 (M3). As presented in Fig 2, the model fit the data well: χ^2^ (59) = 94.98, *p*<0.01; CFI = 0.973; TLI = 0.959; RMSEA = 0.071; 90% RMSEA CI [0.043, 0.096]. The findings revealed that PTG at T1 could significantly predict intrusion symptoms (*β* = -0.24, *p*< 0.01), numbing symptoms (*β* = -0.22, *p*< 0.05), and hyper-arousal symptoms (*β* = -0.22, *p*< 0.01) at T2, whereas PTG at T1 could not significantly predict avoidance symptoms (*β* = -0.15, *p*> 0.05) at T2.

## Discussion

This longitudinal study examined the bidirectional relationships between PTSS and PTG over time among a group of survivors of a natural disaster. Findings revealed that initial PTG at 12 months post-earthquake could relieve subsequent PTSS at 18 months post-earthquake and not vice versus.

Findings of the current study show that the adaptive value of PTG had an effect in reducing PTSS over the time course. From a perspective of PTG as a coping process, for example, PTG has been suggested to be a self-enhancing appraisal [17]. By considering the positive implications or benefits of the event, individuals might consolidate self-esteem, meaning and self-control, which might help in coping with the traumatic event and thus relieve trauma-related distress over time. Davis et al. [16] suggests that PTG as one construal of meaning. By deriving growth from the event and making a benefit attribution for the significance of the event for one’s life, it might help individuals mitigate distress. An alternative explanation is that PTG is positive psychological outcome [8]. As a result of struggling with the traumatic event, the individual might gain growth in several domains, such as a changed perspective on self, others, and the world, which might facilitate adjustment and relieve trauma-related distress, such as PTSS, in the long run. PTG could be both a coping process and an outcome at 12 months post-earthquake, and they showed an overall adaptive effect.

Specifically, this study is original as there have been few studies investigating the predictive effect of PTG on different PTSD symptom clusters. The current findings revealed that PTG at 12 months post-earthquake predicted fewer intrusion symptoms, numbing symptoms and hyper-arousal symptoms, rather than avoidance symptoms, at 18 months post-earthquake. The individual needs for cognitive-emotional processing of the traumatic information following the event, and different PTSD symptom clusters reflect the related but also distinct experiences during the processing [7]. For one thing, PTG might refer to the outcome of cognitive-emotional processing, indicating the development of fundamental positive changes in schema as a result of processing, which could reduce the different PTSD symptom clusters. Additionally, PTG might function in coping in the processing following trauma. By making beneficial attributions and appraisals, individuals might perceive a sense of meaning and hope, which in turn could counterbalance the negative cognitions and emotional distress (e.g., the intrusion, numbing, and hyper-arousal symptoms), but it is stated that there is also the possibility that the individual would use this strategy to avoid the processing of the trauma [9], which might not reduce and may even maintain avoidance symptoms. Taken together, the evidence suggests that early PTG, representing either a coping process or outcome, might play a positive role in reducing later intrusion, numbing, and hyper-arousal symptoms, but the longitudinal relationship between PTG and avoidance symptoms is non-systematic. Additionally, the findings from the current study are consistent with the notion that avoidance and numbing symptoms are distinct in terms of psychopathology and treatment effects [27–29].

Initial PTSS failed to predict the subsequent PTG in the current study. It did not support the assumption that initial distress will facilitate the experience of PTG following a traumatic event [36]. In the current study, the time interval between the two assessment points was six months. It might be that the predictive effect of PTSS on PTG would be more obvious over a longer time interval rather than a short one, as the positive transformation facilitated by the trauma-related distress might take time to occur. For example, in previous studies, initial PTSS had a positive effect on the subsequent PTG [14,15], and the time intervals between two assessment points were over than five years. The required length of time interval appropriate for the detection of a predictive effect of PTSS on PTG is still unclear, and future studies using a multiple-assessment-point design over a relatively long time frame are recommended.

The current study has several limitations. First, we did not investigate the PTSS and PTG at an earlier time, such as at three months or six months post-earthquake. The most affected earthquake area had been struck severely, making the research work relatively difficult to carry out in early stages, but without the information from early stages after the earthquake, we could not describe the longitudinal relationship between PTSS and PTG in a more elaborate way. Second, the majority of the surveyed samples were teachers in the primary and secondary schools most affected in the earthquake area. Although evidence suggesting that SES or educational level would account for PTSS and PTG systematically is not consistent [10,37], future studies are suggest to examine the generalization of results in different samples. Third, the current study examined the numbing symptom cluster measured by IES-R and its relationship with PTG. There were only 2 items measuring numbing symptoms in IES-R, which might contribute to a low reliability (Cronbach’s alpha). Moreover, considering the DSM-V symptomatological criteria for PTSD, it did not measure other dysphoria-related symptoms in the “negative alterations in mood and cognition” symptom cluster defined in the DSM-V. Thus, future studies might use a validated measure of PTSS based on the DSM-V criteria to gain a better understanding of the longitudinal relationship between PTG and different PTSD symptom clusters.

Despite the limitations, the findings have implications for psychological interventions following traumatic events. By using the longitudinal design, the study explored the bidirectional relationships between PTG and PTSS in the first two-year period following a traumatic event, which suggested early psychological growth might play an adaptive role in the alleviation of later PTSS severity. Further studies are suggested to use intervention designs as to test whether integrating the facilitation of PTG into a trauma intervention would relieve the PTSS severity in the long run. Meanwhile, PTG might have different effects on the PTSD symptom clusters. Facilitating PTG can be used for the treatment of several PTSD symptom clusters, such as intrusion, numbing, and hyper-arousal symptoms. However, as the relationship between PTG and avoidance symptoms is non-systematic, it is recommended that counselors take caution in dealing with these two phenomena. One possible suggestion is that facilitating PTG does not mean avoiding the negative trauma-related information or avoiding thinking about it deliberately, but rather helping individuals process the traumatic event cognitively and emotionally, and develop growth during the struggle [38].
